# Chronic inflammation and cancer: potential chemoprevention through nuclear factor kappa B and p53 mutual antagonism

**DOI:** 10.1186/1476-9255-11-23

**Published:** 2014-08-09

**Authors:** Srabani Pal, Ashish Bhattacharjee, Asif Ali, Narayan C Mandal, Subhash C Mandal, Mahadeb Pal

**Affiliations:** 1 Pharmacognosy and Phytotherapy laboratory, Division of Pharmacognosy, Department of Pharmaceutical Technology, Jadavpur University, Kolkata 700032, India; 2 Department of Biotechnology, National Institute of Technology, Durgapur-713209, India; 3 Department of Botany, Visva-Bharati, Santiniketan-731236, India; 4 Division of Molecular Medicine, Bose Institute, Kolkata 700054, India

**Keywords:** Nuclear factor kappa B (NF- *κ*B), Inflammation, Cancer, Phytochemicals, Chemoprevention, Small molecule inhibitors, Toll like receptor (TLR), Tumor necrosis factor (TNF), Lipopolysachharides (LPS), Tumor suppressor p53 (TP53), MDM2, Inhibitor of kB (I *κ*B), Inhibitor of kappaB kinase (IKK)

## Abstract

Activation of nuclear factor-kappa B (NF- *κ*B) as a mechanism of host defense against infection and stress is the central mediator of inflammatory responses. A normal (acute) inflammatory response is activated on urgent basis and is auto-regulated. Chronic inflammation that results due to failure in the regulatory mechanism, however, is largely considered as a critical determinant in the initiation and progression of various forms of cancer. Mechanistically, NF- *κ*B favors this process by inducing various genes responsible for cell survival, proliferation, migration, invasion while at the same time antagonizing growth regulators including tumor suppressor p53. It has been shown by various independent investigations that a down regulation of NF- *κ*B activity directly, or indirectly through the activation of the p53 pathway reduces tumor growth substantially. Therefore, there is a huge effort driven by many laboratories to understand the NF- *κ*B signaling pathways to intervene the function of this crucial player in inflammation and tumorigenesis in order to find an effective inhibitor directly, or through the p53 tumor suppressor. We discuss here on the role of NF- *κ*B in chronic inflammation and cancer, highlighting mutual antagonism between NF- *κ*B and p53 pathways in the process. We also discuss prospective pharmacological modulators of these two pathways, including those that were already tested to affect this mutual antagonism.

## Introduction

Cancer is an extremely complex disease caused by cells that have lost their usual control over growth. The apparent cause of cancer formation may differ case by case, however the basic mechanism is thought to be the following. There are two classes of genes that can control cancer development. Oncogenes and tumor suppressor genes belong to one class, while the other class belongs to the caretaker genes. Healthy cells follow standard rules of growth and proliferation, and have a definitive life span. In contrast, cells with an oncogenic activation undergo much faster cell division with an indefinite life span. Tumor suppressor genes are evolved to inhibit deregulated cell growth. Usually cancer formation ensues when activation and inactivation of an oncogene and a tumor suppressor gene, respectively, occur in a cell at the same time. The caretaker genes control the rate of mutation in the genome. A defective caretaker gene would allow accumulation of mutation in the genome and thus leading to a higher rate of tumor formation. Therefore, cancer formation occurs due to functional defects in multiple genes.

Heredity plays important role in cancer formation. However, it appears to be a small causal factor compared to incidence attributed to the modern lifestyle and environment. Smoking, high calorie diet, obesity, alcohol consumption, chronic infection, exposure to radiation and environmental pollutants are considered to be the major risk factors for cancer formation [1]. In fact about 95% cancer can link modern life style and environment with inflammation as the basic underlying cause [2]. An acute inflammatory response is transient, self regulatory and protects our tissues from infection in a healthy cell. The level of pro-inflammatory cytokines that rises to the peak at the height of the response eventually leads to the production of anti-inflammatory cytokines [3]. Thus an acute inflammatory response is faded off to complete the process of healing. In contrast, chronic or persistent tissue inflammation or irritation is correlated with adverse effects and has long been linked with increasing rate of tumor formation by epidemiological studies [4-6]. Cancer promoted by chronic inflammation (called ‘arbuda’) has been cited in Ayurveda, a form of Indian traditional/alternative medicine ˜5000 years ago. Virchow (in1858) also had observed frequent cancer origination at the site of chronic irritation [4].

## Inflammation as a natural host protective mechanism

As a default mechanism, a cell promptly responds to a tissue injury through activation of innate immunity. The first line of defense is launched by the resident immune cells such as macrophages, dendritic cells, neutrophils, eosinophils, mast cells, natural killer cells present in all tissues. Although neutrophils are the first effectors in acute inflammation, eosinophils are also recruited first sometimes. Monocytes then move to the site of injury and differentiate into macrophages which upon activation release soluble mediators such as interleukin 1 *β* (IL-1 *β*), tumor nicrosis factor *α* (TNF- *α*) interferon IFN- *γ* and chemokines as major effectors of local microenvironment. The mast cells as well affect the local microenvironment at the site of inflammation by secreting several pro-inflammatory mediators including histamines, matrix remodeling proteases to signal migration of different leukocytes (neutrophils, eosinophils, basophils) from adjacent blood vessels to the site of inflammation. Transendothelial migration of a leukocyte is accomplished through distinct sequential steps as follows: Attachment of circulating neutrophil on the vessel endothelium; Stretching and rolling of the attached leukocyte on the endothelium surface; Immobilisation and transendothelial migration of leukocytes to accumulate to the site of inflammation [5,7]. The process of a leukocyte adhesion and rolling on the vascular endothelium surface is mediated by the interaction of the activated E-, and P-selectins on the leukocyte with the intercellular adhesion molecules ICAM1 and ICAM2 on the endothelium. Tight interaction of integrins such as CD11a/ *α*L *β*2, *α*4 *β*1, *α*4 *β*7 *α*L *β*2 with the adhesion molecules VCAM1, MadCAM1 help leukocytes immobilized on the vessel endothelium to migrate to the site of injury [8,9]. Neutrophils are the first leukocytes to migrate to the site of injury followed by monocytes. Leukocytes, at the site of injury, release highly bioactive agents including reactive oxygen species (ROS), nitric oxide (NO), cationic peptides, eicosanoids, different matrix metalloproteases (MMPs) and elastases, clear the cell debris and invading agents through a phagocytosis like process [10,11].

An innate immune reaction (inflammation) associates with appearance of correct profile of chemokines as well as cytokines in an appropriate order at the site of inflammation and is self limiting [12]. A normal inflammatory response is followed by its resolution, and is accompanied by down regulation of proinflammatory cytokines by expression of anti-inflammatory cytokines such as IL-10 [10,13]. Regulatory T cells (Tregs) serve as important source of IL-10 [14]. Patients with mutation in IL-10 receptor develop aggressive diseases, and mice without an IL-10 receptor spontaneously develop inflammation associated colitis correlating with development of colorectal cancer [15]. This function of IL-10 is mediated through inhibition of NF- *κ*B activity involving STAT3 resulting the reduced expression of proinflammatory cytokines like TNF- *α*, IL-6 and IL-12 [16-19]. Many oncogenic factors are cooperatively regulated by STAT3 and NF- *κ*B [20]. Another mode of NF- *κ*B inhibition is mediated through activation of TNFAIP3 gene encoding A20. A20, a ubiquitin editing enzyme with E3 ubiquitin ligase as well as deubiquitinase activities, negatively regulates NF- *κ*B signaling by stepwise deubiquitination and ubiquitination of adaptor molecules associated with TNF- *α* and IL-1 receptors [21]. CYLD, another deubiquitinase negatively regulates NF- *κ*B signaling by targeting several key molecules including NEMO/IKK *γ*, and TNF receptors associated factors TRAF2 [21].

## Correlation between chronic inflammation and cancer

Both epidemiologic as well as clinical studies strongly correlate chronic inflammation with tumor formation [5,12,22,23]. Many individual malignancies are known to originate at the site of chronic infection where the source of infection could range from environmental agents to viral particles. It was estimated earlier that out of 2.2 million cancer cases diagnosed in the world on average more than 15% cases (22% in the developed and 7% in the developing world) can be rooted to infection [24]. There is about 1.2 million new cases of colorectal cancer each year which is majorly caused by chronic inflammation and takes about 60000 lives per year [25]. Persistent inflammation that may result from environmental factors such as exposure to asbestos, smoke, and UV irradiation has been underscored in lung and skin cancer [26,27]. Long term alcohol consumption can cause chronic inflammation in the liver and thus cancer [28,29]. Chronic infection by bacteria can be equally cancerous as well. *Helicobacter pylori* infection has been strongly associated with stomach cancer and MALT-lymphoma in the world [30,31]. Gastric cancer is the second most prevalent cancer in the world [32,33]. Chronic acid reflux is considered as a major reason for esophageal cancer [34].

Schistosomiasis, caused by infection with parasite genus Schistosoma predisposes individuals with increased risks of cancer in internal organs such as bladder and colon [5,35,36]. In fact, schistosomiasis is a socioeconomically devastating disease in developing countries like Asia and Africa [37]. The parasite *Opisthorchis viverrini* infection can lead to cancer in the bile duct, a rare kind of adenocarcinoma [38]. Inflammatory bowel disease such as Crohn’s disease and chronic ulcerative colitis are two good examples of intestinal diseases caused by chronic infection that affect millions of people in the world each year [35,39,40].

Persistent viral infection is thought to be a major cause of hepatocellular carcinoma (HCC). HCC is a third major cause of cancer related death worldwide which claims about 60000 lives each year. About 90% of HCC develops due to chronic infection caused by various agents such as hepatitis B and hepatitis C viruses and, long term alcohol consumption or non alcoholic fatty liver [28,41-44]. Activation of oncogenes is caused by direct insertion of viral DNA such as human papilloma virus (HPV) and Epstein bar virus (EBV), although other mode of actions including degradation of tumor suppressor by viral protein could be critical player in the carcinogenesis process. In cervical cancer E6 protein of HPV degrades p53 tumor suppressor [45]. EBV, a common virus found in human, is conditionally responsible for several cancers such as Hodgkins lymphoma, Burkitt’s lymphoma, nasopharangial carcinoma and lymphoma in the central nervous system (CNS) [5,46,47]. Inflammation was thought to be an essential component in Rous sarcoma virus mediated tumor formation as well [48].

While chronic inflammation is a cause of various cancer as described above, prolong suppression of innate immune response pathway has also been attributed to increased risk for cancer [12,49]. Long term use of antibiotics has been attributed to increased risk of breast cancer [50]. Use of antibiotics has been reportedly associated with increased prostagalandin E2 production catalysed by cyclooxygenases [51]. In fact, mice defective in producing interferon gamma and granulocyte stimulating factor, spontaneously carry low level of inflammation in various tissues that have been correlated with different types of cancer [22,52].

## Role of NF- *κ*B in chronic inflammation and cancer

Role of NF- *κ*B in inflammation was anticipated from the early phase of its discovery; it was activated by various cytokines to subsequently activate the same and other proinflammatory cytokines, chemokines, and adhesion molecules, acute phage proteins, inducible effector enzymes, regulators of cell proliferation and apoptosis. Based on the functional significance associated with innate and adaptive immunity and cell proliferation it is expected that the NF- *κ*B activity is tightly regulated in a cell such as macrophage, dendritic cell or lymphocyte [53].

NF- *κ*B was first discovered as an activity that binds to the *κ*B elements on the immunoglobulin kappa light chain enhancer in the B cells although it occurs, as discovered soon after, in all cell types [54,55]. NF- *κ*B, refers to a group of five structurally related and conserved proteins in mammals i.e., RelA/p65, Rel/cRel, RelB, NF- *κ*B 1/p50, and NF- *κ*B 2/p52. The p50 and p52 are synthesised as larger precursors of p105 and p100, respectively [56]. The family members consist of well defined domain structure correlated with their distinct functions. The N-terminal rel homology domain (RHD) is required for formation of homodimers, or heterodimers between the members that is necessary to execute their transcriptional function [57]. The nuclear localization signal (NLS) resides towards the c-terminus of RHD. A major regulation in NF- *κ*B activity is executed through controlling the trafficking of NF- *κ*B between the nucleus and cytoplasm [53]. In unstimulated cells, the majority of cellular NF- *κ*B is sequestered in the cytoplasm by binding to the inhibitor I *κ*B. I *κ*B, specifically inhibits NF- *κ*B DNA binding function by trapping the NF- *κ*B in the cytoplasm. In I *κ*B-NF- *κ*B complex, the NLS of NF- *κ*B subunits is masked by ankyrin repeats [58]. Except p50/p50 and p52/p52 that are implicated in the repression of transcription, most NF- *κ*B heterodimers act as transcriptional activators [59]. p50/50 and p52/p52 homodimer do not have an activation domain but can activate gene expression by a nuclear mediator [57,60,61].

**Figure 1:**
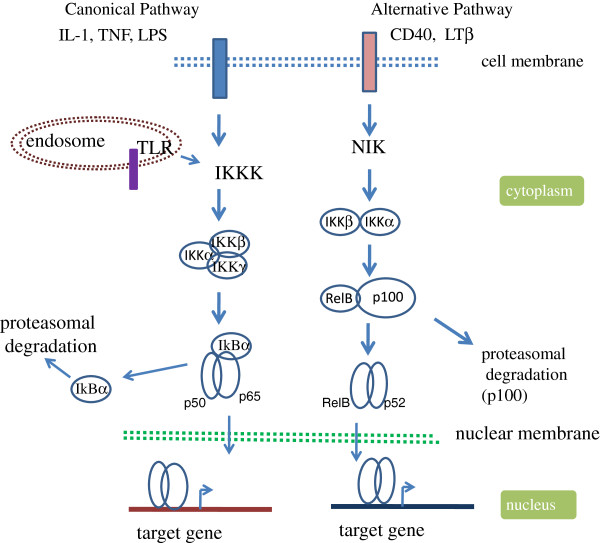
**Activation of NF-*κ*B signaling through canonical and alternative pathways.** Different cytokines and pathogen associated molecules (PAMs) interact with their specific receptors (cytokine receptors:TNF receptor, IL1 receptor; PAMs recognize TLRs present on outer cell membrane (TLR1,-2,-4,-5,-6, and -10) or on the endosomal membrane (TLR3, -7, -8 and -9) in the initial stage of NF- *κ*B activation pathway. The activated receptor recruit the adapter components (not shown) such as Myd88 and TRIF (TIR domain-containing adaptor inducing IFN- *γ*, except TLR3 which utilizes TRIF without Myd88 (Myeloid differentiation primary response protein 88) to transmit the signal through activation of several mediators components including IRAK4 (IL1 receptor associated kinase 4), TRAF6 (TNF receptor associated factor 6), to activate IKKK i.e., MEKK1 (mitogen activated protein (MAP) kinase/extracellular signa l regulated kinase (ERK) kinase kinase 1), MEKK3 and TAK1 (transforming growth factor *β* activated kinase) to act to phosphorylate IKK complex. The activated IKK modifies the inhibitor I *κ*B for its proteasomal degradation. NF- *κ*B is released free to enter into the nucleus for transcriptional activation of the target genes. The canonical pathway is active in innate immunity, inflammation and cell survival; the alternative pathway mediates the humoral immunity.

Activation of NF- *κ*B is mediated by various receptors located on the extra and intracellular membrane. Majority of the knowledge on NF- *κ*B activation pathway came from studying the activation of family of receptors in the class of IL-1 and TNF- *α*. Cytokines such as IL-1 *β*, TNF- *α*, can act in paracrine as well as autocrine manner to activate the NF- *κ*B activity through their cognate receptors on the cell surface. Toll like receptors (TLRs) belonging to the IL receptor family are a group of membrane anchored receptors activate NF- *κ*B in response to specific pathogen associated molecular patterns (PAMPs) including LPS, flageller protein like flagellin, viral double stranded RNA and many pro-inflammatory cytokines (Figure 1) [53,62,63]. Nod (nucleotide-binding oligomerization domain)-like receptors (NLRs) often considered cytoplasmic counter parts of TLRs are a group of 20 receptors activated by bacterial components and toxins to activate NF- *κ*B [64-67]. NLRs localized in the cytosol in mammals are also sensitive to signals created by the presence of dying and injured cells [68,69]. C-type lectin receptors (CLRs) are another group of integral membrane bound receptors present predominantly on the myeloid cells i.e., monocyte, macrophage, granulocyte and dendrtic cells work in conjunction with the TLR and NLR [70]. Triggering receptors expressed on myeloid cells (TREM) proteins are yet another group of cell surface expressed receptors that are involved in the regulation of inflammatory response by leukocytes and differentiation of immune cells [71]. TREM-1 protein expressed on neutrophils and monocytes activates NF- *κ*B in response to bacterial products [72-74].

Upon interaction with a ligand, an activated receptor transmits the signal to I *κ*B kinase (IKK) complex through a mediator kinase [75]. Several kinases have been identified to act on IKK. Transforming growth factor *β*-activated kinase 1 (TAK1) mediated phosphorylation of IKK has been reported in response to several different stimuli such as IL-1 [76] and ubiquitin [77], TNF *α*[78] and LPS [79]. MEKK3 is another kinase that has been implicated in IKK activation in response to certain stimuli [80,81]. Role of autophosphorylation in IKK activation has also been implicated in certain virus-induced NF- *κ*B activation [75,82].

There are two distinct NF- *κ*B signaling pathways: canonical and non-canonical or alternate pathway. The canonical pathway involved in innate immunity is activated by pro-inflammatory stimuli including e.g., TNF- *α*, various interleukins, microbes, and virus related ligands. The alternative pathway on the other hand is associated with adaptive immunity such as lymphoid organogenesis and is stimulated by B cell activating factor (BAFF) [83-85], CD40 ligand, lymphotoxin *β* (LT *β*) [84] and receptor activator of NF- *κ*B ligand (RANKL) [86].

The IKK complex is a critical regulator of NF- *κ*B activation pathway. The I *κ*B kinase (IKK) involved in the canonical pathway is composed of IKK *α* IKK *β* and the regulatory subunit IKK *γ* or NEMO [87-89]. Although the three components of IKK complex is crucial for activation for NF- *κ*B, evidence of NF- *κ*B independent function of IKK *β* also exists [90]. In contrast, the IKK involved in the alternate/noncanonical pathway is composed of IKK *α* and IKK *β*[84]. The alternative pathway deals with the processing of p100 and translocation of p52-RelB dimer, and depends on the phosphorylation of IKK *α* not IKK *β* by NF- *κ*B inducing kinase (NIK) [53,83,84]. The activated IKK phosphorylates conserved residues on both I *κ*B *α* (at ser32 and ser36) and I *κ*B *β* (at ser19 and ser23) [82,91,92]. Phosphorylation induced conformation change tags I *κ*B for recognition by the receptor subunit *β*TrCP for polyubiquitination by specific E3 ubiquitin ligase of Skp1-Cull/F-box (SCF) family [93-95]. The polyubiquitinated I *κ*B undergoes rapid degradation by proteasome, allowing NF- *κ*B such as p50:p65 heterodimer to enter into the nucleus, and bind to the *κ*B motif on the target gene promoters for activation [53,56,96]. Because of the presence of activation domain (AD), only NF- *κ*B containing RelA/p65, RelB and cRel subunits can act as activator. Due to lack of AD, p50 and p52 homo and heterodimer associate with gene repression. The *κ*B bound NF- *κ*B mediates transcription activation function through recruitment of various coactivator including p300/CBP, or PCAF. Different post-transcriptional modifications (PTMs) including phosphorylation, acetylation, ubiquitination, nitrosylation of NF- *κ*B were shown to influence this process [54,97]. For example, phosphorylation of p65 subunit by PKA and or MAPK at ser276 and ser311 by PKC *ζ* stabilizes RelA/p65-CBP interaction [97].

An innate immune response initiated by infection or injury recruits immune cells (such as neutrophils) at the site of injury as a protection mechanism. During this process neutrophils release several highly active antimicrobial agents such as reactive oxygen species, charged peptides, and proteases. Normally, these antimicrobial activities are required for a short period of time as the wound is repaired and self limiting. Secretion of these agents, however, for more than normal period may result in the induced genotoxicity complicated by the constant presence of inflammatory cells. A chronic inflammation associates with a constitutive activation of NF- *κ*B as a result of either an imbalance in the inflammatory signaling network such as inefficient anti-inflammatory mechanism, or persistent infection with pathogens. Furthermore, constant presence of pathogen proteins, activation of a kinase, over expression of a cytokine, and PAMP receptor can drive NF- *κ*B mediated gene expression to promote tumor initiation, progression, invasiveness and its persistence. In fact, the link between NF- *κ*B and cancer was first suspected by its close structural similarity with viral oncoprotein v-Rel [98,99]. Identification of translocation of Bcl3, a member of I *κ*B family in chronic lymphoblastic leukemia (CLL) had at that time reaffirmed the connection of NF- *κ*B with cancer [100]. The significance strengthened when it was discovered that many cancer had constitutively active NF- *κ*B, and that down regulation of NF- *κ*B makes these cells more sensitive to treatments including chemo and radiation therapies [101,102]. Directed inhibition of NF- *κ*B activity regresses tumor growth in mouse models of lung and colitis associated cancers in addition to tumor suppression in xenograft experiments [25,103-105].

Deregulation at any stage in the NF- *κ*B activation pathway can lead to persistent NF- *κ*B activation/chronic inflammation and eventually cancer [12,23,106]. Under this condition, resident immune cells such as macrophages and mast cells constantly monitor the tissue microenvironment, and sense the invading pathogen with pathogen associated molecular pattern (PAMP) through toll like receptors (TLRs) (Figure 1). Thus, TLRs are the first line of sensors for activation of innate immune response. Both epidemiological as well as genetic data link NF- *κ*B activating receptors with cancer [106]. Mutation in TLR cluster TLR1-6-10 in combination with interleukin receptor associated kinase (IRAK) 1 and 4 has been linked with greater risk of prostate cancer [107]. An elevated expression of several TLRs has been associated with different cancer types as well [108,109]. In both mouse and human colorectal cancer TLR4 is over expressed, and mice deficient in TLR4 are insensitive to colon cancer [110]. Thus, TLRs are potentially important drug targets for cancer treatments. As well testing TLR expression profiling in patients is being considered as cancer diagnostic marker [111]. Linkage analysis detected association of 30 different genes including mutation in NOD2, a member of PAMP receptor with the increased incidence of Crohn’s disease [112,113] and inflammatory bowel disease [114]. This has been linked with increased IL-1 *β* production in the inflammatory milieu [23,115]. Several other findings associate DNA mutation with enhanced IL-1 *β* activity, particularly in gastric cancer [116,117]. Notably, abundant IL-1 *β* level in cancer environment associated with increased cancer invasiveness and is considered a good therapeutic target [23,118,119]. Many cancers originate due to paracrine/autocrine expression of cytokines such as IL-1 *β* and TNF- *α* which constitutively activate NF- *κ*B by activation of their cognate receptors [53,120,121].

Abnormal activation of IKK has been implicated in many different cancer types including breast, prostate, brain and colon cancer, melanoma mantle cell lymphoma as recently reviewed elsewhere [75,121]. Vlantis et al. [105] showed that a constitutive overexpression of IKK *β* in the intestinal epithelial cells (IEC) resulted in both inflammation and tumorigenesis. The IKK *β* overexpressing in IECs have elevated levels of pro inflammatory cytokines such as TNF- *α*, IL-1 *β* and various chemokines attracting increased level of infiltrated inflammatory immune cells. The elevated cytokine and chemokine levels modulate Wnt/ *β*catenin signaling leading to the activation of several IEC-associated stem cell factors providing a possible explanation for a switch from inflammation to transformation. Role of Wnt/ *β* -catenin pathway in intestinal cancers has already been implicated [122]. These studies noted IKK *β* as a potential therapeutic target in colorectal cancer. In MALT lymphoma resulting in AP12-MALT1 fusion leads to the constitutive activation of NF- *κ*B through aberrant IKK activity [16]. MUC1 over expressed in several cancers activate NF- *κ*B -p65 through direct interaction with IKK *β* and IKK *γ*[123]. In normal B lymphocytes CARD11 acts as a cytoplasmic scaffolding protein which coordinates the signal mediated- activation of IKK activity. Mutation in the coiled-coil domain of this protein resulting in the gain of function in the form of constitutive activation of IKK as observed in the diffuse B cell lymphoma [124]. In human T cell leukemia virus 1 (HTLV1) mediated transformation of host cell, the HTLV-1 protein Tax constitutively activate NF- *κ*B by direct interaction with the IKK complex [125].

Thus far a constitutively active mutation in the NF- *κ*B protein itself has not been found. Bcr-Abl, a tyrosine kinase, supports acute lymphoblastic leukemia (ALL) and chronic myelogenous leukemia (CML) by inducing NF- *κ*B function through enhancing nuclear translocation of NF- *κ*B. A defective I *κ*B leads to constitutively active NF- *κ*B in the Hodgkin cells [126]. An overexpression of tissue transglutaminase (TG) underlies a basis of many aggressive and drug resistant cancers including pancreatic ductal carcinoma [127]. Mann et al. have shown that elevated levels of TG drives constitutive NF- *κ*B overexpression in these cancers [128].

While NF- *κ*B promotes oncogenic potential of cells by driving expression of genes encoding prosurvival and proliferative functions, it is also antagonistic with tumor suppressor such as p53 [106,129]. For example, NF- *κ*B target gene MDM2, an ubiquitin E3 ligase drive p53 for proteasomal degradation [130]. P53 can also antagonise NF- *κ*B by competing for cellular p300/CBP and vice versa [131]. Several other tumor suppressors including ARF (p14^ARF^) and PTEN may also antagonize transcriptional activation and function of NF- *κ*B [132-135]. Putative tumor suppressors LZAP (LXXLL/leucine-zipper-containing ARF-binding protein), transcription elongation factor A like 7 (TCEA7) and CHFR (check point with forkhead and ring finger domains) can also antagonize with NF- *κ*B activity by interfering with the transcriptional activities of RelA subunit [136-138]. While cross-talk activities of NF- *κ*B with the tumor suppressors is finely controlled during normal cellular homeostasis, a deregulation in any of the control points can result in its deregulation adding to tumor promoting capability of NF- *κ*B [133,139].

## Role of p53 in inflammation: p53 and NF- *κ*B antagonizes each other’s function

Tumor suppressor p53 is one of the most extensively studied proteins due to its functional association with the maintenance of genomic integrity. In fact, in more than 50% cancers the p53 protein is either absent or nonfunctional due to various other reasons. p53 is termed ‘the guardian of genome’ for the major function it plays in protecting cells from transformation and genomic mutation in response to a various stressors including DNA damage, oxidative stress, and oncogene activation through activation of cellular process like cell cycle arrest, apoptosis, or senescence [140,141]. Activation of p53 also associates with induction of other important processes including autophagy, angiogenesis, cell migration, and differentiation [141].

A healthy cell constitutively maintains p53 at low level [142]. Usually, in an unstressed condition p53 is constantly ubiquitinated by MDM2, an E3 ubiquitin ligase to channel it to proteasomal degradation pathway [143]. MDM2, a p53 transcriptional target gene, is upregulated as p53 level goes up; an elevated MDM2 in its turn checks the normal low level of p53 in the cell by funneling extra level of p53 to proteasome for degradation. Thus p53 and MDM2 constitutively controls each others activity in a normal healthy cell. MDM2 executes this function as a heterodimer with a structurally related protein MDM4 (MDMX) [144,145]. MDM2 controls the activity of MDM4, also a p53 target gene, through its E3-ubiquitin ligase activity. Thus p53 activity is tightly controlled by two autoregulatory loops; one through MDM2 and another through MDM4. The association of p53 with MDM2 and MDM4 is disrupted with phosphorylation by various enzymes such as DNA-PK, ABL, ATR, ATM and CHK -all activated by genotoxic stressors [146]. Phsophorylation of MDM2 at ser17 and MDM4 at Tyr99 by DNA-PK [147] and c-ABL [148], respectively, drives dissociation of MDM2, and activation of p53. MDM2 was reported to inhibit p53 transcription function by blocking p53 surface that interact with the basal transcription factors such as TFIIE [149]. ATM and CHK1 kinases induced by genotoxic stress signals activate p53 by driving dissociation of MDM2 and MDM4 through phophorylation [150]. In contrast, activation of AKT kinase function in cancer stabilizes MDM2 and MDM4 interactions resulting in inhibition of p53 activity [151,152]. In addition to simple dissociation of p53 from its negative regulator MDM proteins, removal of ubiquitin moiety from p53 by ubiquitin proteases can lead to its stabilization as well [142].

In fact, the major part of p53 tumor suppressor activity is explained by its transcriptional activation function. The active p53 undergoes extensive post-translational modifications including phosphorylation, acetylation, monoubiquitination, and neddylation guided by specific stress signals. A particular posttranslational modification targets p53 to a subset of its target gene promoters [153,154]. Furthermore, p53 executes its tumor suppressor activity in tissue specifc manner [155,156]. A specific posttranslational modification may allow p53 to interact with a partner protein, or a promoter DNA sequence contributing to tissue specific gene activation [154,157].

Depending on the nature of signal p53 induces a set of target genes resulting in a definite cell fate. For example, expression of p21/WAF1, 14-3-3 *σ* and Cdc25c genes links with reversible cell cycle arrest at different stages of cell cycle [158-161]. Elevated expression of genes such as Puma (p53-upregulated modulator of apoptosis), Noxa, Bax (Bcl-2 associated X protein), Apaf-1 (Apoptotic protease activating factor-1) results in cellular apoptosis [162]. Expression of Pai-1 (plasminogen activator inhibitor type 1) and p21 associates with a state of an irreversible cell cycle arrest or replicative senescence [163,164]. Induction of DRAM (Damage-regulated autophagy modulator) leads to autophagy [165]. In addition to its role in transcriptional activation, p53 is also implicated in transcriptional repression of many genes involving various mechanisms [166].

**Figure 2:**
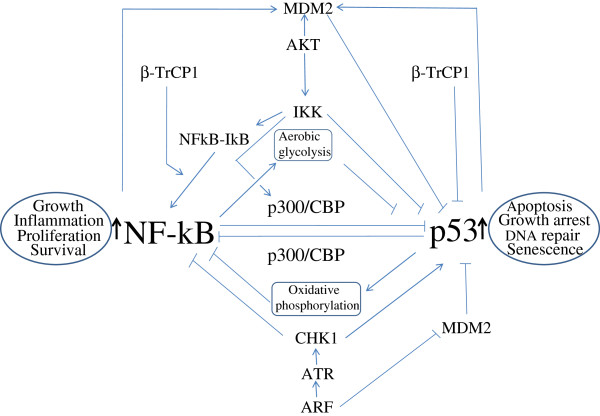
**NF-*κ*B and p53 antagonises each others activity.** Various mediators involved in the pathways are indicated. In addition, p53 and NF- *κ*B can inhibit each other by direct physical interaction through their multimerization domain. Eventually, the effect of activation NF- *κ*B pathway prevents the activation of p53 pathway and vice versa. The detail is described in the text. The black colored upward arrows adjacent to NF- *κ*B and p53 indicate activation of these transcription factors. MDM2: mouse double mute 2; *β*-TrCP1: beta transducing repeat containing protein1; ARF: alternate reading frame of INK4/ARF locus; ATR: ATM-Rad3 related; CHK: check point kinase; I *κ*B: inhibitior of kB; IKK: inhibitor of kappaB kinase.

In principle p53 should be induced in an inflammatory condition, however, this is not the case normally. In fact most cells have mechanisms to suppress p53 when NF- *κ*B is activated to tilt the situation in favor of the cellular transformation process. Through various mechanisms, NF- *κ*B may cripple cellular p53 activities (Figure 2) [129,167]. MDM2-p53 network discussed earlier can be interrupted by NF- *κ*B. First, MDM2 is a NF- *κ*B target gene. NF- *κ*B can suppress p53 levels by upregulating the MDM2 expression mediated through Bcl3 [168], or IKK *β*[169]. It has been shown that DNA damage induced upregulation of Bcl3 can inhibit p53 through upregulation of Hdm2 gene transcription [168]. Bcl3, a member of I *κ*B family, helps activate NF- *κ*B in a cooperative manner in cellular proliferation [168]. It has been shown that the presence of an active IKK *β* can block doxorubicin induced p53 mediated apoptosis and this event requires IKK *β* kinase function which is mediated by NF- *κ*B through MDM2 [169].

Activation of AKT (a serine threonin kinase) by PI3 kinase in response to growth factor, plays important role in cancer cell growth and proliferation (mediated by induction of anti-apoptotic mechanism). AKT favors prosurvival pathway by inhibiting p53 and supporting NF- *κ*B activities at the same time. It inactivates proapoptotic Bad protein through phosphorylation at three positions (at ser112, -136 and -155) which prevents its binding with anti-apoptotic protein Bcl-x_L_[170,171]. Bad, a member of Bcl2 family proteins, is p53 target gene [171]. AKT can block p53 accumulation and apoptosis by augmenting ubiquitin ligase activity of MDM2 activity through phosphorylation [172]. AKT mediated phosphorylation of MDM2 at ser166 and ser186 facilitates nuclear entry of MDM2 and faster degradation of p53. AKT also activates NF- *κ*B through phosphorylation of both IKK *α* and IKK *β* subunits of IKK that facilitates nuclear translocation by I *κ*B phosphorylation and degradation [173]. Furthermore, AKT was reported to activate IKK *α* in response to PDGF [174], and IKK *β* in response to TNF *α*[175].

IKK *β* can also block cellular p53 stability through direct post translational modification while activating NF- *κ*B through phosphorylation of I *κ*B. It was shown that p53 can be phosphorylated at ser366 and potentially at ser362 by IKK *β* facilitating its ubiquitination by *β*-TrCP, a member of SCF^b-TrCP^ E3 ligase complex and proteosomal degradation [176].

ARF tumor suppressor known as p14^ARF^ and p19^ARF^ in human and mice, respectively, induced upon oncogene such as E2F activation, has been shown to facilitate p53 activation [134]. ARF induces p53 accumulation by inhibiting p53 association with MDM2/Hdm2 [177]. In addition, ARF was shown to block NF- *κ*B function. ARF inhibits NF- *κ*B activation by inducing association with histone deacetylase, HDAC1 [134]. RelA/p65 subunit when phosphorylated at thr505 by Chk1 kinase was shown to associate with HDAC1. ARF induction activates Chk1 kinase via induction of ATR kinase [178].

An effect of p53 products on cell fate can be neutralized by the products of the NF- *κ*B target genes; NF- *κ*B induced antiapoptotic genes including Bcl2 can abrogate the pro-apoptotic functions of PUMA, and Noxa induced by p53 [179].

In addition, Ikeda et al. showed that NF- *κ*B/RelA and p53 can inhibit each other’s activity through direct physical interaction with multimerisation domains of the transcription factors [180].

A major interference of p53 activity by NF- *κ*B is mediated by their use of a common coactivator p300 and CBP for their optimal activity in response to a stress signal [181,182]. It has been shown that through the depletion of limited pool of cellular p300/CBP, NF- *κ*B competes out and thus represses p53 function [183]. IKK *α* mediated phosphorylation of a specific amino acid residue on CBP makes it preferentially utilized by NF- *κ*B instead of p53 [131,184].

It was shown that ectopic expression of p53 inhibits NF- *κ*B function in a unique way; expression of p53 enhanced NF- *κ*B DNA binding but blocked it transactivation function [185]. NF- *κ*B and p53 cross action has been associated with the regulation of actin cytoskeleton function and integrin signaling both of which play important role in tumor progression. It has been shown that in the absence of p53, STAT3 is constitutively activated in an NF- *κ*B-dependent manner which regulate lamellipodia formation via integrin signaling [186].

Glucose metabolism is another aspect which is inversely controlled by p53 and NF-*k*B. Absence of p53 enhances NF- *κ*B activity through activation of IKK kinase and thereby increase the rate of glycolysis. It was shown that absence of p53 upregulates expression of high affinity glucose transporter GLUT3 as observed in tumor microenvironment [187]. In contrast, activation of p53 has been linked with reducing the expression of several glycolysis regulating factor including glucose transporters [188]. In addition to slowing down glucose uptake, p53 induction was shown to promote oxidative phosphorylation [189,190].

Finally, an inverse correlation between NF- *κ*B and p53 has been demonstrated in model animals. First, it was shown that mice bearing p53 null homozygous mutation was born normal from embryonic stem cell but prone to tumor formation during development starting at 6 month of age [191]. Later it was shown that p53-/- mice had relatively higher level of proinflammatory cytokines and chemokines and reduced level of oxidation products, while those mice ectopically expressed p53 carried lower level of those cytokines compared to their respective controls. Komarova et al. also shown that LNCaP cells transduced with p53 suppressor element expressed much lower level of proinflammatory markers [192]. Furthermore, treatement of cells with inducer of p53 caused inhibition of NF- *κ*B target genes expression [103,104]. Recently, Natarajan et al. shows that transduction of cells with NF- *κ*B activating polypeptides isolated by genetic selection called as NF- *κ*B activating selectable peptides (NASP), reduced p53 accumulation [106].

## Small molecule NF- *κ*B inhibitors as chemopreventive agents

The presence of constitutively active NF- *κ*B apears to be a common underlying factor in inflammation related cancer (responsible for constitutive induction of different prosurvival genes such as antiapoptotic genes: Bcl2, Bcl-x; proangiogenic gene: VEGF; genes encoding metastatic and invasion activities: MMPs). Consistently, both clinical and epidemiological data suggest strong chemopreventive potential for NF- *κ*B inhibitors. Chemoprevention refers to use of chemicals, including even food supplements to prevent the development and progression of cancer.

Given the significance of NF- *κ*B function, many laboratories have undertaken prime interests to deliver a specific and potent inhibitor of NF- *κ*B pathway (Table 1). A series of small molecules acting on single as well as multiple steps in the NF- *κ*B signaling pathway have been reported, some of which are in various stages of clinical trials [58,129,193,194]. The nature of these molecules includes protein/antibodies, small peptide, small RNA/DNA, and small molecular weight chemicals. Development of inhibitors targeting basically every possible step in the NF- *κ*B signaling pathway is underway including molecules interfering with the receptor-ligand interaction, interference of the adapter with the activated receptor, activation of IKK and subsequent phosphorylation leading to proteasomal degradation of I *κ*B, nuclear translocation of NF- *κ*B, binding of NF- *κ*B with the target gene promoter, and interaction of the activator with the co-activator (for detail list of inhibitors see [194]). Normally the outcome of activation of NF- *κ*B is determined by the cross-talk among the parallel signaling pathways such as p53, PTEN, and p38 MAPK [133].

**Table 1:** **Selective inhibitors of NF-*κ*** **B signaling pathways that inhibits IKK activity and I** ***κ*** **B** ***α* phosphorylation and/or degradation**

**Molecule**	**Point of inhibition**	**References**
BMS-345541 (4(2-Aminoethyl)amino-1,8-dimethylimidazo(1,2-a) quinoxaline) and 4-amino derivatives	IKK *α* and IKK *β* kinase activity	[210]
2-amino-3-cyano-4-aryl-6-(2-hydroxy-phenyl)pyridine derivatives	IKK *β* activity	[211]
Acrolein	IKK *β* activity/p50 DNA binding	[212]
1-[2-cyano-3,12-dioxooleana-1,9(11)-dien-28-oyl] imidazole	IKK *β* activity	[213]
Dihydroxyphenylethanol	IKK *β* activity	[214]
MLB120 (small molecule)	IKK *β* activity	[215]
SC-514 (small molecule)	IKK *β* activity	[215,216]
Thienopyridine	IKKb activity	[217]
Amino-pyrimidine derivative	IKK activity	[58]
Benzoimidazole derivative	IKK activity	[58]
Butein	IKK *β* activity	[218]
Beta-carboline	IKK activity	[219]
Berberine	IKK *β* activity	[220]
IMD-0354	IKK *β* activity	[221]
PS-1145 (MLN1145)	IKK *β* activity	[222]
17-Acetoxyjolkinolide B	IKK activity	[223]
CML-1	IKK activity	[224]
CT20126	IKK activity/NIK	[225]
Furonaphthoquinone	IKK activity	[226]
3-Formylchromone	IKK *β* activity/p65 DNA binding	[227]
Indolecarboxamide derivative	IKK activity	[58]
(Amino) imidazolylcarboxaldehyde derivative	IKK activity	[58]
Imidazolylquinoline-carboxaldehyde derivative	IKK activity	[58]
ML120B	IKK activity	[228]
Pinitol	IKK activity	[229]
PMX464	IKK activity	[230]
Pyrazolo[4,3-c]quinoline derivative	IKK activity	[58]
Pyridooxazinone derivative	IKK activity	[58]
N-(4-hydroxyphenyl) retinamide	IKK activity	[231]
Thalidomide (and thalidomide analogs)	IKK activity	[232]
Salubrinal	IKK activity/degradation	[233]
GS143	Blocks I *κ*B ubiquitylation	[208]
Delphinidin	Phosphorylation	[234]
Digitoxin	Phosphorylation	[235]
Dihydrotestosterone	Phosphorylation	[236]
Kaempferol	Phosphorylation	[237]
Tomatidine	Phosphorylation	[238]
Allylpyrocatechol	Degradation	[239]
Clomipramine/imipramine	Degradation	[240]
Glucosamine (sulfate or carboxybutyrylated)	Degradation	[241]
Losartan	Degradation/NF- *κ*B expression	[242]
Pectenotoxin-2	Degradation	[243]
Sevoflurane/isoflurane	Degradation	[244]

A major point of regulation in the NF- *κ*B activation cascade involves signal induced release of NF- *κ*B from I *κ*B in the cytoplasm (Table 1). A signal activated IKK phosporylates I *κ*B *α* which is targeted by *β*-TrCP mediated ubiquitination and subsequent proteosomal degradation. *β*-TrCP, the F-box component of SCF-E3 ubiquitin ligase, through its WD40 domain recognizes the destruction motif of I *κ*B *α* in phosphorylation dependent manner [93-95]. *β*-TrCP inhibitors which target I *κ*B *α* degradation in the NF- *κ*B activation pathway blocking the release of NF- *κ*B for entry into the nucleus have shown a great promise in the treatment of disease such as multiple myeloma (MM) [58,195]. MM cells are very sensitive to a proteasome inhibitor like bortezomib due to two reasons: a constitutively activated NF *κ*B and an over dependence on proteasome activity [196]. Bortezomib (or Velcade) was the first drug as proteasome inhibitor (reversible inhibitor) approved by US food and drug administration for treatment of MM [197-199] (Table 2). By definition, however, proteasome inhibitors will be nonspecific because cellular proteins recognized by the proteasome regulating various signaling pathways will also be sensitive to this treatment. For example, many proteasome substrates not related to NF- *κ*B pathway having pro-apoptotic and cell cycle regulatory activities will be stabilized and thus enhance bortezomib activity [200]. These players include program cell death regulator (PDC4) [201], myeloid cell leukemia 1 (Mcl-1) [202] and cell cycle division cycle homologue 25A (CDC25A) [203,204]. Furthermore, bortezomib treatment stabilizes a factor like *β*-catenin, also a *β*-TrCP substrate. In fact, an elevated level of *β*-catenin has been observed in several cancers including colorectal and liver cancer [58,205-207]. However, GS143, a proteasome inhibitor was reported to degrade *β*-catenin. It was reported to block I *κ*B *α* degration, not its phosphorylation [208]. MG-132 was reported as another specific, potent, reversible, and cell-permeable proteasome inhibitor with Ki= 4 nM. It was shown to reduce the degradation of ubiquitin-conjugated proteins in mammalian cells and in strains of yeast (with membrane permeable ability) by the 26S complex without affecting its ATPase or isopeptidase activities. MG132 can also inhibit NF- *κ*B activation with an IC50 of 3 *μ*M. MG-132 by blocking I *κBα* degradation has been shown to potently inhibit TNF- *α*-induced NF- *κ*B activation, interleukin-8 (IL-8) gene transcription, and IL-8 protein release in A549 cells [209].

**Table 2:** **Small molecules modulators of both NF-*κ*** **B and p53 signaling pathways**

**Name of compounds**	**Structure of the compounds**	**Molecular weights**	**Signaling pathways affected**	**References**
R-Roscovitine		354	a) Abrogates induction of NF- *κ*B by preventing I *κ*B kinase (IKK) kinase activity, NF- *κ*B /p65-536ser phosphorylation b) Induces p53 by disrupting p53-MDM2 interaction	[245-247]
Flavopiridol		438	a) Blocks steps in the NF- *κ*B activation pathway such as I *κ*B *α* kinase and p65 nuclear translocation, and p65-529-ser phosphorylation b) Reversibly activates p53 by inhibiting MDM2	[248-250]
Nutlin-3		581	a) Inhibits NF- *κ*B targets ICAM1 and MCP1 transcription in p53 dependent manner. b) Potent inducer of p53 by inhibiting MDM2 interaction	[251,252]
Curcumin		368	a) Inhibits IKKkinase, promoinflammatory gene promoters such as TNF-2 *α*, COX-1, COX-2 b) p53 activation by inhibiting MDM2 c) modulation of other signaling pathways	[253-256]
Quinacrine		473	a) Inhibits both constitutive and inducible form of NF- *κ*B irrespective of the p53 status. b) Activates p53	[104,167]
Curaxin (CBL 137)		338	a) Simultaneously suppress NF- *κ*B both basal and inducible states and activate p53 by targeting FACT b) Inhibits NF- *κ*B, activates p53 by inhibition of FACT complex	[103,167,257]
Benfur		322	Inhibits NF- *κ*B activity which is predominantly dependent on p53-mediated pathway	[258]
Resveratrol		228	Inhibits NF- *κ*B /p65 and p53 transcriptional functions by deacetylation of specific residues	[259-262]
Pifithrin- *α*		367	Activation of NF- *κ*B through blockade of p53-p300 interaction	[263,264]
Bortezomib		384	Block of NF- *κ*B and stabilization of p53 by inhibiting E3 ubiquitin ligases	[265]

Here only parents compounds were mentioned. Compounds are in dervatizations for better functional efficacies are described/referred in text/table.

Although 35% MM patients respond to bortezomib, it was a great success for treating MM and mantle cell lymphoma patients. Nevertheless, acquisition of resistance to bortezomib in MM patients is common with reported over expression of or mutation in the *β*5 subunit of the proteasome catalytic core complex. This has led to the development of second generation proteasome inhibitor carfilzomib. Carfilzomib, reported to be a potent and an irreversible inhibitor of the proteasome, that drives higher rate of apoptosis has completed phase III clinical trial [193,266-268]. Marizomib is another proteasome inhibitor with increased potency and sustained inhibitory activity compared to bortezomib [269-271]. Marizomib, isolated from marine actinomycete *Salinspora tropica* is the first irreversible proteasome inhibitor from natural sources, had been in phase I clinical trial to assess its efficacy against solid tumors and treatment of refractory multiple myeloma [272,273]. Marizomib blocks all three catalytic sites of 26S proteasome. In addition, several newly developed proteasome inhibitors including CEP-18770, MLN-9709, ONX-0912, NPI-0052 are currently in various phases of clinical trials for their efficacy against myeloma and solid tumors [199,274-277].

Thalidomide and its analogues known as immunomodulatory drugs (IMiDs), have anticancer as well as anti-inflammatory effects. Recently, these agents, including IMiD CC-5013 and IMiD CC-4047 [278], have shown promise in clinical trials for the treatment of different cancers. Among several different hypotheses, the inhibition of NF- *κ*B activation has been proposed to explain the therapeutic activity of thalidomide and related drugs [279]. In endothelial cells, thalidomide blocks the degradation of I *κ* B- *α* by inhibiting IKK- *β*, which is consistent with its role in inhibiting cytokine-induced NF- *κ*B activation [279]. The inhibitory effect of thalidomide on TNF- *α* and H_2_O_2_-induced NF- *κ*B activation is also seen in other cell types, including T lymphocytes, and myeloid and epithelial cells [280]. IMiD-induced apoptosis in multiple myeloma cells is associated with downregulation of NF- *κ*B DNA-binding activity, as well as the reduced expression of NF- *κ*B-dependent proteins [281]. Hence a major part of the immunosuppressive effects of thalidomide might be due to inhibition of NF- *κ*B activation.

Cyclopentenone prostaglandins (cyPGs) are naturally occurring prostaglandin metabolites which inhibit NF- *κ*B activation or activity [282]. This effect could be partly due to the ability of cyPGs to activate the peroxisome proliferation-activated receptor- *γ* (PPAR- *γ*), which was shown to antagonize NF- *κ*B transcriptional activity [283]. The treatment of peritoneal macrophages with the cyPG 15-deoxy- *Δ*12, 14-prostaglandin J2 (15d-PGJ2) inhibits the expression of inducible nitric oxide synthase (iNOS), as well as NF- *κ*B activity in a PPAR- *γ*-dependent manner. However, cyPGs can directly inhibit activation of NF- *κ*B pathway by blocking IKK- *β* activity [284].

Nonsteroidal anti-inflammatory drugs (NSAIDs) are known to act as protective agents in cancer treatments, in particular inflammation associated cancer in model animals and humans. Although NSAIDs are popularly known as the blocker of prostaglandin (PGE) synthesis by COX-2, they have considerable NF- *κ*B inhibitory activities unrelated to COX inhibition. COX-2, the rate limiting enzyme for production of PGE from arachidonic acid is NF- *κ*B inducible [285-287]. Indomethacin and related compounds sulindac, sulindac sulfide and sulindac sulfone, were shown to block IKK *β* kinase activity in colon cancer and other cell lines [288]. Aspirin and salicylate act as competitive inhibitors of IKK- *β* for its ATP binding pocket and thus prevent I *κ*B phosphorylation and NF- *κ*B activation [289]. These NSAIDs are also shown to block NF- *κ*B mediated expression of VCAM-I and ICAM-I induced by TNF- *α*[288]. Salicylate is also reported to prevent expression of endothelial leukocyte adhesion molecule and leukocyte transmigration through endothelial monolayer [290]. Sulphasalazine is another NSAID that is widely used to treat inflammatory bowel disease. This compound is cleaved following oral administration to 5-amino-salicylic acid (5-ASA) and sulphapyridine. The treatment of human colonic epithelial cells with sulphasalazine, but not 5-ASA or sulphapyridine, inhibits NF- *κ*B activation through blocking I *κ* B phosphorylation and degradation in response to TNF- *α*, LPS or phorbol esters [291]. However, in a more recent study, 5-ASA was shown to block NF- *κ*B activation by inhibiting both IKK- *α* and IKK- *β* kinase activity in mouse colonic cells [292]. Mesalamine, a related aminosalicylate, can block phosphorylation of p65 without affecting I *κ*B degradation [293]. These results indicate that these agents can block the NF- *κ*B activation pathway at multiple steps.

‘The development of selective IKK or NF- *κ*B inhibitors has been undertaken by several pharmaceutical industries (Table 1). Yet, no potent IKK- *α*-specific inhibitors have been described so far. Several compounds, which are under development, can inhibit IKK- *α* kinase activity in the low micromolar range, although these agents were initially identified as IKK- *β* inhibitors. The unique role of IKK- *α* in the alternative pathway, important for B-cell mediated responses, and the recent demonstration of the auxiliary role of IKK- *α* in the classical pathway, indicate that IKK- *α* might be an important target for therapeutic intervention in autoimmune diseases and cancer [84,294-296]. By comparison, the development of specific IKK- *β* inhibitors has progressed rather rapidly. Although most IKK- *β* inhibitors reported so far are still in preclinical stages of development, a number of novel small-molecule inhibitors of IKK- *β* have been described. For example, SPC-839, a member of a series of quinazoline analogues developed by Celgene [297-299], is one of the more extensively studied IKK- *β* inhibitors. SPC-839 inhibits IKK- *β* with an IC50 of 62 nM, and has a 200-fold selectivity for IKK- *β* over IKK- *α* (IC50 = 13 *μ*M). Several groups have indicated the inhibition of IKK- *β* activity by *β*-carboline derivatives [222,300,301]. PS-1145, which was developed from a *β*-carboline natural product inhibits the IKK complex with an IC50 of 150 nM, blocks TNF- *α*-induced I *κ*B phosphorylation and degradation in HeLa cells [222]. Another molecule that inhibits IKK- *β* is BMS-345541, which is an imidazoquinoxaline derivative [210,302]. BMS-345541 shows greater than tenfold selectivity for IKK- *β* (IC50 =0.3*μ*M) over IKK- *α* (IC50 =4*μ*M). In addition, several other compounds like ureidocarboxamido thiophenes [303-305], 2-amino-3-cyano-4,6,-diarylpyridines [306-308], anilinopyrimidine derivatives [309], a group of optically active pyridine analogues [310], and a group of related pyridyl cyanoguanidines [311,312] have been reported as nanomolar-range selective inhibitors of IKK- *β* kinase activity. Bay 11-7082 (BAY) is an inhibitor of *κ*B kinase (IKK) that has pharmacological activities that include anticancer, neuroprotective, and anti-inflammatory effects. BAY-11-7082 selectively and irreversibly inhibits NF- *κ*B activation by blocking TNF- *α*-induced phosphorylation of I *κ*B- *α* without affecting constitutive I *κ* B- *α* phosphorylation [313].

Dehydroxymethylepoxyquinomicin (DHMEQ), derived from the structure of an antibiotic epoxyquinomicin C is a novel NF- *κ*B inhibitor. DHMEQ could be qualified as a candidate for a new chemotherapeutic agent against human hepatoma [314]. It can also enhance antitumor activities of taxanes in anaplastic thyroid cancer (ATC) cells. DHMEQ blocks the nuclear translocation of NF- *κ*B. Inhibition of NF- *κ*B by DHMEQ creates a chemosensitive environment and greatly enhances apoptosis in taxanes-treated ATC cells in vitro and in vivo [315].

I3C/DIM Indole-3-carbinol (I3C) is a glucosinolate when given orally is converted to diindolylmethane (DIM) and other oligomers catalyzed by stomach acid. DIM is the predominant active agent and that I3C is a precursor. Combinatorial treatment of I3C/DIM with N-acetyl-S-(N-2-phenethylthiocarbamoyl)-l-cysteine (PEITC-NAC) and myo-inositol (MI) caused marked reductions in the activation of Akt, ERK and NF-kB in lung tumor tissues and thereby demonstrated the promise of combination therapy using I3C/DIM for the chemoprevention of lung carcinogenesis in smokers [316].

## Small molecule inhibitors of NF- *κ*B from natural sources

From the beginning of civilization, herbal medicines, fruit and vegetables have been used in the disease treatment and well being of humans. It is also believed that plant based treatments are without side effects. Interestingly, many phytochemicals with NF- *κ*B inhibitory activity with cancer cell type specificity have been isolated (for a detail review on inhibitors see [194]) (Table 3). These compounds can make cancer cells sensitive to apoptosis or inhibit the expression of genes responsible for growth, proliferation and the metastasization of cancer cells. Different groups isolated battery of compounds from different traditional and ethnic medicinal plant sources against cancer cells of different tissue origins such as multiple myeloma cells (U266 line and MM.1 line), prostate cancer cells. Mode of action of these compounds is increasingly becoming clear. Many of these compounds inhibit both inducible as well as constitutively active NF- *κ*B activities. Compounds with diverse specificity have been isolated with specificity towards the IKK or IKKK (IKK kinase), I *κ*B *α* stability, p65 translocation or DNA binding in the NF- *κ*B activation pathway. Celastrol isolated from *Celastrus orbiculatus* inhibit various stimuli -induced phophorylation of I *κ*B *α* and its degradation. Importantly, celastrol is found to block IKK function and IKK *β* activity in dose dependent manner [225]. Epicatechin present in green tea, for example, prevents constitutive NF- *κ*B activity facilitating apoptosis by preventing p65 translocation to nucleus [317]. Apigenin, a flavonoid present in most fruits and vegetables blocks p65 phosphorylation by inhibiting IKK function [318,319].

**Table 3:** **NF-*κ*** **B inhibitors from dietary/natural products**

**Name of phytochemicals**	**Dietary/natural source**	**Chemical nature**	**Mechanism of action**	**References**
Celastrol	Root extracts of *Celastrus Tripterygium wilfordii*(Thunder god vine), and *Celastrus orbiculatus,Celastrus regelii*	A quinone methide triterpenoid	a) Blocks cytosolic I *κBα* degradation and nuclear translocation of RelA.	[247,320]
			b) Blocks IKK function and IKK *β* activity.	[225]
Epicatechin	Green Tea, Cocoa, Grapes	Pentahydroxyflavane	a) Blocks constitutive NF- *κ*B activity by blocking p65 nuclear translocation.	[317]
			b) Inhibits NF- *κ*B DNA-binding activity	
Epigallocatechin-3-gallate (EGCG)	Green Tea	Ester of epigallacatechin and gallic acid (a type of catechin)	a) Inhibits IKK activation, I *κBα* degradation, and NF- *κ*B activation. In addition, EGCG inhibited phosphorylation of the p65 subunit of NF- *κ*B.	[321]
			b) Prevents nuclear translocation of p65	[322]
Apigenin	Parsley, Thyme, Peppermint	Trihydroxyflavone	a) Blocks p65 phosphorylation by inhibiting IKK function	[318]
			b) Suppress NF- *κ*B translocation to nucleus and inhibits I *κBα* phosphorylation and degradation	[323]
Xantholhumol	Hops, Beer	Prenylated chalconoid	Inhibits NF- *κ*B through suppression of I *κBα* phosphorylation	[324]
Genistein	Soybeans, Fava beans	4,5,7-trihydroxyisoflavone	a) Blocks activation of NF- *κ*B concomitant with degradation of I *κBα*	[325]
			b) Exerts its inhibitory effect on NF- *κ*B signaling through Akt pathway	[326]
Capsaicin	Chilli pepper	8-methyl-*N*-vanillyl-6-nonenamide	a) Blocks I *κ*B- *α* degradation and nuclear translocation of p65 b) Inhibits NF- *κ*B activity by blocking I *κ*B- *α* degradation and phosphorylation	[258]
Boswellin	Produced by plants in the genus *Boswellia*	Pentacyclic triterpene	Inhibits constitutively activated NF- *κ*B signaling by inhibiting IKK activity	[327]
Escin	*Aesculus hippocastanum* (the horse chestnut).	Pentacyclic triterpene	Inhibits TNF-induced IKK activation, I *κBα* phosphorylation and degradation	[328]
Sesamin	Isolated from the bark of *Fagara* plants and from sesame oil	Lipid soluble lignan	a) Blocks NF- *κ*B activation by blocking I *κ*B- *α* degradation and phosphorylation	[329]
			b) Down regulates both constitutive and inducible NF- *κ*B activation. Suppress p65 phosphorylation and nuclear translocation	[330]
Luteolin	Celery, broccoli, green pepper, parsley, thyme	A polyphenol flavonoid [2-(3,4-Dihydroxyphenyl)- 5,7-dihydroxy-4-chromenone]	Inhibition of NF- *κ*B activity by accumulation of ROS	[331]
Parthenolide	occurs naturally in the plant feverfew (*Tanacetum parthenium*)	Sesquiterpene lactone	Inhibits of NF- *κ*B both indirectly by inhibiting IKK, and directly by modifying p65 at a key cysteine residue in its activation loop	[332]

An administration of antioxidant *N*-acetyl-L-cysteine (NAC) suppresses LPS-induced NF- *κ*B activity [333]. In another study it is shown that vitamin C inhibits TNF- *α*- and IL-1 *β*-induced IKK phosphorylation of I *κ*B- *α* and subsequent NF- *κ*B DNA binding in endothelial cell lines [334]. Further studies reveal that dehydroascorbic acid (DHA), which is the oxidized form of ascorbic acid, suppresses TNF- *α*-induced NF- *κ*B activation by the direct inhibition of IKK- *β* kinase activity independent of p38 MAPK [335]. It should be noted that antioxidants can also inhibit the activity of other components of NF- *κ*B signaling pathways, including TNF receptors and the proteasome, without any direct effect on IKK [336].

Various molecules have been isolated that target IKK include xantholhumol from hops [255], epigallocatechin-3-gallate from green tea [337], genistein from soy [338], capsaisin from chilli pepper [339], boswellin from *Boswellia serrata*[340], and sulforaphane from cruciferous vegetables [341] (Table 3). Aggarwal and colleagues have isolated and functionally characterized a group of compounds targeting the NF- *κ*B activation pathways [121]. Some of these include a pentacyclic triterpenoid escin isolated from horse chestnut whose extract is used as traditional medicine in China. Escin inhibits activation of IKK activity [328]; Sesamin isolated from sesame seed inhibits IKK kinase [329]; Bharangin, a diterpenoid quinonemethide isolated from *Premna herbacea***,** an Indian medicinal plant also has similar activity [342].

Other compounds from natural/dietary sources, with NF- *κ*B inhibitory activity and are currently in preclinical or clinical trials include Luteolin and parthenolide. Luteolin, a polyphenol flavonoid, has been reported to sensitize colorectal cancer cells to TNF-induced apoptosis through suppression of NF- *κ*B. Accumulation of ROS induced by luteolin plays a pivotal role in suppression of NF- *κ*B and potentiation of JNK to sensitize lung cancer cells to undergo TNF-induced apoptosis [331]. Zerumbone, isolated from subtropical ginger has been shown to inhibit angiogenesis through inhibition of NF- *κ*B in gastric cancer cell line [343]. Parthenolide is a major active component of the herbal medicine feverfew (Tanacetum parthenium), which is conventionally used in Europe to treat inflammatory diseases such as fever, migraine, and arthritis [344]. Parthenolide has been shown to inhibit growth or induce apoptosis in a number of tumor cell lines [345-348]. Many mechanisms are postulated to be involved in the antitumor effect of parthenolide, including inhibition of NF- *κ*B [347]. It has also been shown that parthenolide sensitizes cancer cells to various apoptosis-inducing agents mainly through inhibition of NF- *κ*B [349].

Several compounds have been isolated from traditionally used plants that have inhibitory activities against multiple components of NF- *κ*B activation pathway [121]. Curcumin from common Indian spice turmeric and resveratrol from grape have drawn a lot of attention because of their activity against multiple signaling pathways. Curcumin inhibits IKK- mediated phosphorylation of I *κ*B as demonstrated in Burkitt lymphoma cells. Curcumin prevents NF- *κ*B/p65 phospohorylation at serine 536 and its acetylation through p300 inhibition (Table 2) [350]. Burkitt lymphoma cells expressing wild type Bax protein undergo apoptosis by curcumin treatment. Curcumin sensitizes Bax negative Burkitt lymphoma cells to TNF-related apoptosis inducing ligand (TRAIL) which activates apoptosis through the extrinsic pathway [351]. As a transcriptional regulator, it inhibits production of eicosanoids prostaglandin E (PGE_2_) and 5-hydroxyeicosatetraenoic acid (5-HETE) and is in phase II clinical trial for different malignancies including pancreatic colorectal cancers [352,353]. Recently, curcumin has been described to block proteasome function in cell based assays [354]. Curcumin was described to block the chymotrypsin like activity of the catalytic core of rabbit 20S and cellular 26S proteasome. In a recent study curcumin was described to target different components of ubiquitin proteasome pathway [355]. Resveratrol, a plant polyphenol found in different fruits (grapes, berries etc) came to lime light in 1997 for its anti tumor activity tested against various tumors such as myeloid, breast, and prostate [356]. Resveratrol has been shown to down regulate the expression of many antiapoptotic genes. Resveratrol inhibits constitutive activation of NF- *κ*B by inhibiting both IKK as well as p65 phosphorylation [357,358]. Preparation of resveratrol deratives for better afficacy is underway [259].

## Small molecule activators of p53 pathway

A significant cancer population carries a non-functional p53 tumor suppressor gene. The p53 functional defect can result due to the overexpression of its negative regulator MDM2 and or MDMX, or mutation in the gene body producing p53 non-functional for not properly folding. An elevated level of MDM2/MDMX prevents proper accumulation of p53. As conceivable, a mis-folded p53 can be dysfunctional in DNA binding or interaction with itself or another protein. Some population of p53 associated cancer is known to carry a nonsense mutation in their p53 gene. Given the strong mechanistic link between p53 and cancer, many small molecule activators of p53 with potential pharmacological values have been reported with some targeting the p53 defects discussed above. See [157] for review on p53 activators.

The first non-peptide small molecule that demonstrated the possibility of inhibiting the p53–MDM2 interaction was 4,5 dihydroimidazoline (nutlin; Roche) [252]. The crystal structure of nutlin 3a when bound to MDM2, provided the template for the design of better inhibitors such as the benzodiazepinedione family of compounds [359], chromenotriazolopyrimidine [360], terphenyls [361,362] and chalcones [363,364]. At the same time, structure-based screening of compounds combined with molecular modelling enabled the development of a new class of inhibitors like the spiro-oxindole based molecules. This led to the identification of MI219, which has a subnanomolar affinity for MDM2, can taken orally. MI-219 has good pharmacokinetic and pharmacodynamic (PK/PD) properties and increases the level of p53 as well as the p53 target genes CDKN1A and MDM2 [365]. The same group also developed a series of diastereomeric spiro-oxindoles such as MI888 which is a potent MDM2 inhibitor (Ki =0.44 nM) with a superior pharmacokinetic profile and enhanced in vivo efficacy [366]. Simultaneously, other drugs that have been reported so far include pyrazole and imidazole compounds [367], imidazole-indoles [368], isoindolinones [369] pyrrolidinones such as PXN822 [370,371], piperidines [372], spirooxindoles [373] and the sulphonamide NSC279287 [374].

Restoration of p53 activity by inhibition of the p53-MDM2 interaction has been considered an attractive approach for cancer treatment. Currently, the most advanced MDM2 inhibitors include RG7112 (Roche), RO5503781 (Roche), MI773 (Sanofi), DS3032b (Daiichi Sankyo), which are at various stages of Phase I clinical trials [157]. After the discovery of RG7112, which was the first small-molecule p53-MDM2 inhibitor in clinical development, many groups reported the discovery and characterization of a second generation clinical MDM2 inhibitor, with superior potency and selectivity like RG7388 [375], RO5353 and RO2468 (two new potent, selective, and orally active p53-MDM2 antagonists [376], AM-8553 (a potent and selective piperidinone inhibitor of the MDM2-p53 interaction.with promising potential for clinical development) [377] and AMG 232 (an extremely potent MDM2 inhibitor with remarkable pharmacokinetic properties and in vivo antitumor activity) [378]. Recently a putative small-molecule inhibitor of p53-MDM2 interaction, pyranoxanthone, was discovered using a yeast p53 transactivation assay based approach [379]. Pyranoxanthone mimicked the activity of known p53 activators, leading to p53 stabilization and activation of p53-dependent transcriptional activity. A novel and promising lead structure for the development of anticancer drugs as MDM2-p53 interaction disruptor, 3-benzylideneindolin-2-one derivative, was also identified recently using both pharmacophore- and structure-based approaches [380].

A few preclinical lead compounds which restore the activity of mutant p53 also recently completed Phase I trials. PRIMA1 (a 2,2bis(hydroxymethyl)-3quinuclidinone and its structural analogue PRIMA-1 ^Met^ (APR246, developed by Aprea) have been shown to restore mutant p53 activity *in vitro* and *in vivo*[381-383]. PRIMA-1 ^Met^ seems to lead to the formation of covalent adducts on mutant p53-R175H and p53-R273H proteins, but its exact mechanism of action has yet to be fully understood [384] and needs further investigation. PRIMA-1 ^Met^ also seems to be able to restore the function of mutant p63 (a p53 homologue) [385,386]. Other therapeutics that target mutant p53 via various mechanisms have been described; for example, NSC176327 and RETRA disrupt the binding interactions between mutant p53 and p73, whereas MIRA1 eliminates mutant p53 [371,387].

Two nonsense mutations at position R196X and R213X in p53 gene have been linked with large number of cancer patients. Thus, small molecules and drugs that promote the read-through of nonsense codons in p53 could provide a novel approach for treating tumours carrying this type of mutation [388,389]. A recent study showed that treatment of the human tumour cell line HDQP1, which contains a homozygous nonsense mutation at codon 213 (CGA-TGA), with the read-through-promoting aminoglycoside antibiotic G418 led to a dramatic increase in the level of *TP53*mRNA and full-length p53 protein [390]. This restored full-length protein might re-establish the p53-MDM2 feedback loop, so a combination of G418 and nutlin may be especially effective. There are a number of potential read-through drugs in development because of high demand due to the association of p53-readthrough mutation with genetic diseases like Duchenne muscular dystrophy [157].

Cell-based screens for activators of the p53 pathway have identified large numbers of compounds which have unknown targets and incompletely defined mechanisms of action. In a few cases, the mechanisms by which these compounds activate p53 have been determined. These include the small molecule RITA (to target p53 itself) [391] (cyclin-dependent kinase (CDK) inhibitors such as roscovitine [246,392], RNA polymerase inhibitors such as actinomycin D [393], exportin 1-binding compounds such as leptomycin B and KPT-330 [394], the NEDD1 (neural precursor cell expressed developmentally downregulated protein 1) ligase inhibitor MLN4924 [395] and sirtuin inhibitors such as the tenovins [396]. All of the compounds described above (with the possible exception of RITA) act on targets that will affect other pathways in addition to the p53 pathway and will therefore require careful evaluation in preclinical models.

Several MDM2 inhibitors derived from natural products have also been identified, such as the prenylated xanthones *α*-mangostin (from the fruit of *Garcinia mangostana L.*) and gambogic acid (from the resin of *Garcinia hanburyi*) [379,397]. These molecules are thought to bind in a manner similar to nutlins and open up future prospect for the development of new classes of non-toxic inhibitors. As natural products, they also offer potential as chemopreventive anticancer agents without side effects. Marine organisms are now also being considered as a potential resource for a variety of lead compounds. For example, the siladenoserinols, derived from the marine invertebrate family Didemnidae, were recently reported to inhibit the p53–MDM2 interactions [398].

## Small molecule modulators of both p53 and NF- *κ*B pathways

Cancer is a complex disease and results due to defect in multiple signaling pathways [399]. Thus drug targeting multiple pathways is thought to be a better option in cancer therapy than a single component [102,129]. In fact, combinatorial therapy or drugs that affect multiple signaling pathways or multiple steps in one pathway are expected to be more effective in cancer treatment. Critical roles of NF- *κ*B in cancer include induction of genes that prevent cell death and promote cell proliferation, and antagonize tumor suppressor p53. Several compounds simulteneously targeting more than one pathways such as inhibition of NF- *κ*B while activating p53 have been considered very promising chemopreventive agents with some already are in the various phases of clinical trials (Table 2). Anti-malaria drug quinacrine was identified to have dual activities of inhibiting NF- *κ*B and activating inactive cellular p53 by cell based assays. Quinacrine as well as other derivatives of aminoacridines are DNA-damage mimetic, non genotoxic with excellent anticancer therapeutic potential as tested in mouse xenograph models. This is notable because anticancer agents like cisplatin induces p53 by formation of covalent-DNA adducts. Quinacrine inhibits both constitutive and inducible form of NF- *κ*B [104]. This NF- *κ*B inhibitory activity has been shown to be independent of the p53 status of a cell because quinacrine strongly inhibits NF- *κ*B irrespective of their p53 status [104]. The p53 inducing activity of quinacrine which acts in micromolar range is thought to be based on its ability the planar molecule to intercalate between the adjacent DNA base pairs with its side chain interacting with the minor groove [103]. Quinacrine can also target several signaling pathways such as PI3K/AKT/mTOR in addition to NF- *κ*B and p53 [257]. Curaxins are newly discovered prospective anticancer drugs that at nano molar concentration simultaneously down regulate NF- *κ*B while activating the p53 [400]. Like quinacrine, curaxins activate p53 without detectable damage in the cellular DNA. These carbazol structure based compounds intercalates between the DNA base pairs while its side chain binds the minor group of nucleic acids. Mechanistically, the anticancer property of this group of compounds may rely on the trapping of the transcription elongation factor FACT (facilitates chromatin transcription) on the chromatin making it unavailable for transcription of the NF- *κ*B target genes that are dependent on FACT [401,402]. FACT upon binding to DNA-curaxin complex induces casein kinase 2 (CK2). Activated CK2 phosphorylates p53 (at serine 392) to drive p53 for interaction with FACT. Curaxins apparently does not interfere with other aspects in the NF- *κ*B activation pathway such as nuclear translocation of p65/p50 heterodimer. FACT, originally implicated as histone H2A/2B chaperone and a heterodimer of SSRP1 and Spt16 is utilized by RNA polymerase II for transcription of nucleosomal DNA [403]. Lippard and colleagues earlier identified FACT complex by its ability to bind cisplatin modified DNA [404].

R-Roscovitine (seliciclib, CYC202), a 2, 6, 9 substituted purine analogue is another small molecule that prevent tumor growth by targeting multiple signaling pathways simultaneously. It blocks constitutively active NF- *κ*B activity while activating the p53 tumor suppressor and in phase II clinical trial for its anticancer property. It was shown that R-Roscovitine abrogated TNF- *α* and IL-1 mediated induction of NF- *κ*B by preventing I *κ*B kinase (IKK) kinase activity [245]. The purine analogue also blocks p65 phosphorylation at ser536 by IKK that is required for nuclear translocation and chromatin remodeling function. At the level of transcription, R-Roscovitine represses transcription of NF- *κ*B target genes MCP-1, ICAM-1, COX2 and IL-8 [245,392]. R-Roscovitine originally isolated as cyclin dependent kinase inhibitor (CDK), induces p53 by disrupting p53-MDM2 interaction. R-Roscovitine was shown to downregulate MDM2 both at the level of protein and mRNA [247]. Retinoblastoma protein phosphorylation and activation of MAP kinase are other identified functions of R-Roscovitine [405].

Flavoridol (Table 2), an inhibitor of multiple CDKs (CDK1, -2, -4 and -7) as well simultaneously inhibits NF- *κ*B and activates p53 is considered as an agent in combinational chemotherapy. A semisynthetic flavonoid, flavoperidol blocks TNF *α* induced NF- *κ*B induction by blocking steps in the NF- *κ*B activation pathway such as I *κ*B *α* kinase and p65 nuclear translocation, and cyclin D1 transcription [250]. Flavoperidol inhibits cellular transcription through targeting positive transcription elongation factor (pTEFb), a kinase targeting the serine 2 residue of largest subunit of RNA polymerase II [249,406]. Flavoperidol was shown to reversibly activate p53 by inhibiting MDM2, and sensitize cells towards apoptosis [406,407].

Nutlin was isolated as the first specific interrupter of p53-MDM2 interaction and is considered as a potent inducer of p53. It was also shown to inhibit activation of NF- *κ*B target genes such as ICAM-1 and MCP-1 through p53 dependent manner in lung cancer cells [251,252]. While p53 can induce MDM2 expression; MDM2 on the other hand can influence p53 activity in multiple ways including inhibition of transactivation function, removing the tumor suppressor out of nucleus, and directing it to the proteasomal degradation pathway through its own E3 ubiquitin ligase activity [408]. Grasberger and colleagues also isolated benzodiazepinedione based MDM2 inhibitors with similar functional properties [359,409]. A number of compounds (peptide/small molecules) are being studied that is thought to stabilize p53 activity inducing proper folding correcting readthrough error in the p53 gene to activate p53 functions that include for example, PRIMA-1 [383] or RTC13 [410], respectively. Compounds that are doubly inhibitory against MDM2 and MDM4 have also been under investigation [146]. These later group of compounds should be used along with MDM2 inhibtors for effective p53 accumulation. In principle these compounds would be able to work like nutlins mechanistically for their anticancer properties.

Synthetic derivative of benzofuran lignan (Benfur) has a reported antitumor activity. Benfur-mediated cell death is partially regulated by the inhibition of NF- *κ*B activity but it is predominantly dependent on p53-mediated pathway [258]. Benfur potentially inhibits NF- *κ*B DNA binding activity by inhibiting I *κ*B *α* and thereby blocking the nuclear translocation of p65 in both Jurkat and U937 cells. Benfur treatment was shown to increase p53 level by inhibiting Sp1 binding on the MDM2 promoter [258].

## Conclusions

NF- *κ*B, a central regulator of innate immune response, normally is activated in a time dependent manner as a host protection mechanism. It has been now established by numerous independent studies that a persistent long term activation of this factor is tumorigenic and blockade of the activities of an inflammatory mediator regress tumor progression as well as its aggressiveness. Studies from various independent laboratories have already pinpointed some mediators in the NF- *κ*B activation pathway that undergo aberrant regulation in various cancers. Studies from many laboratories have firmly established a reverse correlation of activation between the NF- *κ*B and p53 pathways highlighting a prospective avenue in cancer chemotherapy. Several small molecules of natural or synthetic origin many of which target multiple signaling pathways including NF- *κ*B and p53 apparently hold a great promise to move the cancer treatment and management in the desired direction. In this direction, various potential pharmacological agents have been isolated that activate p53 in tumor cells with high potency at very low concentration. Most of these small molecules appears to have at least part their mode action through inhibition of NF- *κ*B. Conversely, it is conceivable that the anticancer activity of many NF- *κ*B inhibitors is partly due to their ability to induce p53 in cancer cells. Efforts are already underway to find small molecules that will with higher specificity rectify the defect in the pathways to suppress NF- *κ*B activity. Clearly, more studies are needed to provide us with more insightful understanding of the mechanism of deregulation and the underlying cause. Given the role of NF- *κ*B in innate immunity and cancer, a desired objective would be to achieve the ability to turn off and on the function of NF- *κ*B with high precision and as needed.

## Competing interests

The authors declare that they have no competing interests.

## Authors’ contributions

SP, NCM, SCM, and MP conceived of- and designed the review. SP, AB, NCM, SCM, and MP wrote the manuscript. AA contributed to critical reading and comments of the manuscript. All authors read and approved the final manuscript.
